# Social uncertainty in the digital world

**DOI:** 10.1016/j.tics.2024.02.005

**Published:** 2024-04

**Authors:** Amanda M. Ferguson, Georgia Turner, Amy Orben

**Affiliations:** 1 Medical Research Council (MRC) Cognition and Brain Sciences Unit, University of Cambridge, Cambridge, UK

**Keywords:** Bayesian inference, digital affordances, social media, social uncertainty

## Abstract

The social world is inherently uncertain. We present a computational framework for thinking about how increasingly popular online environments modulate the social uncertainty we experience, depending on the type of social inferences we make. This framework draws on Bayesian inference, which involves combining multiple informational sources to update our beliefs.

## Main text

Our social world is rife with **uncertainty** (see Glossary) [1]. To make appropriate choices and thrive as social beings, we must constantly find ways to reduce this uncertainty [2]. Cognitive research has explored multiple human strategies to achieve this [3]. However, it has consistently overlooked the extent to which social lives now play out online: the average American spends 9 h online each day, and nearly half of young people are online 'almost constantly' [4].

Cognitive researchers need to scrutinise the implications of this shift, as the **online social environment** differs fundamentally from its offline counterpart (Table 1). One prominent and underexplored difference is the systematic change to **social uncertainty** in online relative to offline environments. For example, many social media platforms enable asynchronous communication – messages can be easily viewed without an immediate response from the receiver. Receiving no response to a message is a highly uncertain social cue: your friend could be upset with you, or they could be asleep.

**Table 1:** Characterising the online social world

Digital affordance	Related digital features	Description
Anonymity	Usernames, 'throwaway' accounts/multiple accounts	Many online environments are designed to be used anonymously (e.g., Reddit), and concealing one's identity in online communication is much simpler than doing so in offline communication
Asynchronicity	Read receipts, asynchronous messaging	Digital communication allows for time lapses within a single conversation in a way that in-person communication typically does not
Editability	Photo and video filters and editing tools	Online messages and content can be rehearsed and edited before posting in a way that in-person communication cannot
Personalisation	Recommendation algorithms	The degree to which online content is tailored to fit the identity, preferences, or expectations of the viewer
Publicness	Privacy settings, screenshots, sharing, retweeting	Some online environments allow accessibility of information to potentially enormous audiences, far beyond what is accessible in offline environments
Quantifiability	Likes, reactions, followers, friends	Many online environments allow countable social metrics that are often ambiguous and effusive in offline environments, such as 'followers', 'friends', and 'likes'

Such changes to social uncertainty in online environments are transforming our social landscape, as well as the cognitive strategies required to make sense of it. Cognitive researchers have the unique opportunity to apply and adapt pre-existing approaches to understand this change. Such work will help to address pressing questions about the impact of digitalisation on individuals and society.

## A Bayesian account of social uncertainty

**Figure 1:**
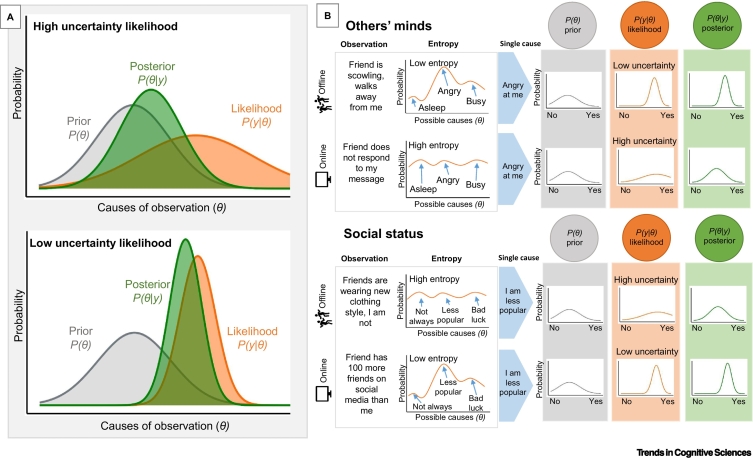
Bayesian belief updating in different environments. (A) Bayesian inference with high (top) and low (bottom) uncertainty likelihoods. Predictions about potential causes of an observation (*θ*) are generated by weighing the probabilities (likelihood, orange) of new evidence (*y*) against one's existing beliefs (prior, grey) to form an updated set of predictions (posterior, green). Bayes’ equation specifies how the prior and likelihood are optimally combined to produce the posterior belief and, specifically, how the value and uncertainty of the prior and likelihood influence the value and uncertainty of the posterior. Uncertainty is represented by the standard deviation of each probability distribution. Wide (or 'flat') distributions, representing many probable causes, are the most uncertain. Narrow distributions, representing a smaller range of probable causes, are the least uncertain. Crucially, the posterior produced by the combination of the prior and likelihood is influenced by the uncertainty of the likelihood. There are specific implications for the way that a change in uncertainty of the likelihood affects posterior beliefs when modelling the probability distributions as normal distributions. Priors become more influential when uncertainty of the likelihood increases. In addition, given the same prior expectations, and the same value of the likelihood (i.e., inferred cause, represented by the location of the peak of the distribution), a more uncertain likelihood (top) means the posterior (i) decreases in certainty (flatter distribution), and (ii) shifts in value towards the prior and away from the likelihood, compared with more certain likelihoods (bottom). (B) Changes to social uncertainty about others’ minds (top) and social status (bottom) in offline versus online environments. An observation evokes a set of possible causes that are considered by an individual, each with their own probability distribution. Entropy measures uncertainty in terms of the probabilities of each cause. When making inferences about the content of others’ minds (top), there is similar evidence for each possible cause (high entropy) for the online versus offline observation. Online social information is more uncertain than offline information (orange, likelihood), as indicated by the wider likelihood distribution for the online observation. Given the same prior expectation (grey), this difference in uncertainty of information results in different beliefs (green, posterior) about an underlying cause of the observation. The inference that a friend is 'angry at me' is both more certain and closer to the peak of the likelihood in the offline versus online environment. When making predictions about social status (bottom), the reverse is true. For the online observation, there are fewer similarly plausible underlying causes (low entropy). The prediction 'I am less popular' is both more certain and closer to the peak of the likelihood in the online versus offline environment. For easier comparability, all examples relate to social rejection, which can be conveyed in a range of ways for minds or status and online or offline.

Our brains use observations to infer the underlying causal structure of our surroundings [5]. These inferences allow us to make predictions about the future informing our beliefs and actions. Uncertainty describes the precision with which a prediction can be made, given the available information [1]. We can be uncertain about the consequences of an observation, how we should act, or the reliability of our information source (**source reliability**) [6]. This uncertainty directly impacts the inferences that we can draw about the world around us [3] (Figure 1A). The amount of uncertainty in a given situation can be quantified with Shannon’s **entropy** formula [2], which measures the probabilities of each possible underlying cause (Figure 1B). Entropy is lowest when a single cause is very likely, and it increases when there are multiple plausible causes.

While we deal with uncertainty constantly, the social world represents a particularly difficult inference problem [1]. Social interactions have inherently higher entropy than non-social experiences, as uncertainty about the experiences of others compounds uncertainty about our own. To reduce this uncertainty and decide what to do next, we must adjudicate amongst the possible underlying causes for our social observations [2,3]. The mathematically optimal solution to this is specified by Bayes’ equation, which dictates that one should update beliefs in proportion to the uncertainty of available information [5]. Accumulating evidence suggests that humans employ a strategy that is well characterised by such **Bayesian inference** when solving inferential problems [5].

Navigating the social world in this way requires us to make inferences about many social domains, such as the content of others’ minds and social status [3]. Deductions about others’ minds include questions about their character (e.g., are they kind?), current emotional state (e.g., angry?), and goals (e.g., selling something?). By contrast, deductions about social status concern how people relate to each other, for example, individual popularity or community structure (e.g., which groups exist and how separate are they?).

## Online social uncertainty: a framework

The designs of digital environments, governed by their so-called **digital affordances** (Table 1), enable online social experiences to significantly deviate from their offline equivalents. The affordances of many social media platforms – such as asynchronicity of communication and quantification of feedback (e.g., 'likes') [7] – change the uncertainty inherent to social interaction. Specifically, they modulate the uncertainty of incoming social information (affecting the Bayesian 'likelihood'), and this has downstream consequences for our inferences ('posteriors'): when the likelihood is more uncertain, we rely more on existing **priors** (Figure 1A). Importantly, these existing beliefs vary across individuals: for example, prior beliefs about the validity of online sources will moderate the overall perceived uncertainty of online information [6].

How a given online environment changes the incoming likelihood of social information, and in turn the corresponding posterior, depends both on its affordances and the social inferences being made. Compared to offline environments, social information about the content of others’ minds is often less certain on social media (Figure 1B). Whereas offline interactions benefit from a plethora of social signals – such as vocal tone and facial expression – interactions on social media do not. Conversely, social information about status may be more certain on social media. While offline signals about social status can be abstract [8], quantified social feedback, public friendship networks, and group chats on social media clearly delineate social status and boundaries between groups.

## Individual differences in social uncertainty

Our framework can be used to understand individual differences in experiences with social media, and to generate novel predictions. People who find inferences about others’ minds more difficult regardless of context, such as those with autism spectrum disorder (ASD), may be less impacted by increased uncertainty about others’ minds online. For people with ASD, the increase in uncertainty online may simply mirror the uncertainty already experienced offline. Furthermore, given that uncertainty is aversive [2], relative gains in certainty about group structure may make online environments more predictable and desirable. This aligns with evidence suggesting that individuals with ASD appear to especially benefit from online relationships [9].

For those who are particularly motivated to maintain relationships, such as adolescents whose developmental goals are inherently social (e.g., identity development, peer inclusion), shifts in social uncertainty will be especially salient [1]. Altered social uncertainty online might therefore explain some of this group’s unique sensitivity to the negative effects of social media on well-being [10]. Computational studies have shown that uncertainty is associated with increased exploration (particularly in adolescents [11]) as we attempt to reduce uncertainty through learning. On social media, increased uncertainty about others’ minds might therefore lead to increased rumination, which is the repetitive exploration of previous actions and experiences. Conversely, as beliefs are updated according to more certain evidence (Figure 1A), social status could increasingly determine the shape of our beliefs. This has implications for the development of depression, which is characterised by a perception that one has low social value and persistent negative beliefs about the causes and consequences of low status [12].

## Concluding remarks

The digitisation of social life shows no signs of slowing [4]. Researchers investigating how humans understand the social world must therefore consider the substantial differences between online and offline environments. One critical difference explored here is the change in social uncertainty. Online environments, such as social media, substantially alter the available social information. However, these changes are not uniform across social domains: uncertainty about the state of others’ minds increases, while uncertainty about social status decreases.

When exposed to highly uncertain online information about others’ minds (e.g., their thoughts, emotions, traits), people may lean increasingly on more certain online social information, such as social status, to make social inferences. This shift has important implications for social and cognitive development, interpersonal and group dynamics, and mental health – yet it has been largely unexamined. Future theoretical and empirical work addressing this question could substantially accelerate our understanding of cognition and social processing in the digital age while also informing best-practice design and regulation of online environments.

## Declaration of interests

The authors declare no conflicts of interest.

## Glossary

**Bayesian inference**: the process by which we generate predictions about the underlying causal structure of the world by weighing the probabilities (likelihood) of new information against one's existing beliefs (priors) to form an updated set of beliefs (posteriors).
**Digital affordance**: latent action possibilities that emerge from digital features and user perceptions of them.
**Entropy**: a measure of uncertainty based on a set of known event probabilities. Entropy is highest when these probabilities are all equal. Entropy is lower when a smaller number of possible underlying causes become more likely than others, and lowest when one possible cause is completely certain.
**Online social environment**: a digital platform that is composed of a set of digital affordances and allows for users to interact with one another. For example, social media platforms such as Facebook and WhatsApp are both online social environments despite each having different digital features and affordances. Shortened to 'online environment' throughout the text.
**Prior**: a distribution of probabilities that define one's belief in the absence of new evidence.
**Source reliability**: the extent to which we trust an information source. Estimations of uncertainty of incoming information can be understood as a joint estimation of hypothesis reliability and source reliability. Belief in the reliability of a source will affect the extent to which one views information from that source as uncertain.
**Social uncertainty**: uncertainty about one's own actions and outcomes is multiplied by uncertainty about the actions and outcomes of others.
**Uncertainty**: describes the precision with which a prediction can be made, given the available information.
